# Toxicity and Efficacy of Thirty Insecticides Against *Thrips flavus* in Northeast China: Laboratory, Semifield, and Field Trials

**DOI:** 10.3390/insects16040405

**Published:** 2025-04-11

**Authors:** Tianhao Pei, Long Wang, Yijin Zhao, Shusen Shi, Yu Gao

**Affiliations:** 1 College of Plant Protection, Jilin Agricultural University, Changchun 130118, China; 2 Dalian City Investment Asset Management Co., Ltd., Dalian 116021, China; 3 Key Laboratory of Soybean Disease and Pest Control, Ministry of Agriculture and Rural Affairs, Changchun 130118, China

**Keywords:** insecticide, toxicity, thrips, soybean pest, chemical control

## Abstract

In soybean fields across Northeast China, *Thrips flavus* Schrank (Thysanoptera, Thripidae) populations are increasing, posing a significant threat to soybean production. The aim of this study was to evaluate the toxicity, insecticide efficacy, and field performance of thirty insecticides against *T. flavus*. Laboratory bioassays revealed that fenthion (LC_50_ = 2.26 mg/L), sulfoxaflor (LC_50_ = 4.28 mg/L), cyetpyrafen (LC_50_ = 4.94 mg/L), and imidacloprid (LC_50_ = 6.16 mg/L) exhibited the highest toxicity against *T. flavus*. Pot experiments were subsequently conducted to assess insecticide efficacy. Seven days after application at the highest tested concentration, the insecticide efficacy of fenthion, sulfoxaflor, chlorantraniliprole, bifenazate, and malathion achieved 100% control efficacy. The four insecticides were selected for field trials based on their high toxicity and insecticide efficacy. Seven days after application, the field efficacy of fenthion at 11.25 g a.i.·hm^−2^, sulfoxaflor at 1.19 g a.i.·hm^−2^, cyetpyrafen at 8.10 g a.i.·hm^−2^, and imidacloprid at 11.25 g a.i.·hm^−2^ exceeded 80%. Thus, these four insecticides hold strong potential for integrated management of *T. flavus*. Overall, the findings provide a valuable reference for developing chemical control strategies against this pest.

## Simple Summary

*Thrips flavus* Schrank (Thysanoptera, Thripidae) has recently emerged as a predominant pest in soybean fields across Northeast China. Currently, no insecticides are explicitly registered for thrips control in soybean fields in China. Thus, the objective of this study was to identify and evaluate insecticides with potential for inclusion in integrated pest management (IPM) strategies targeting this pest. Thirty insecticides, all registered in China for thrips control on crops other than soybean, were investigated for their toxicity and insecticide efficacy against *T. flavus*. Based on the results, four insecticides, fenthion, sulfoxaflor, cyetpyrafen, and imidacloprid, were selected for further field trials. These compounds exhibited the highest toxicity and insecticide efficacy, with field control efficiency exceeding 80%, indicating strong potential for use in future IPM strategies. The findings provide a basis for developing chemical control measures against *T. flavus* in soybean production.

## 1. Introduction

Soybean is a crucial crop globally, playing a critical role in food production and the agricultural economy [1]. However, its cultivation is continually threatened by various pests that can substantially reduce both yields and quality. Among these pests, thrips have recently emerged as a growing concern. The term “thrips” broadly refers to members of the order Thysanoptera, encompassing over 6000 species [2,3]. Thrips exhibit diverse feeding habits, including phytophagous, mycophagy, and predation. Some species can also transmit plant pollen. Phytophagous thrips can cause severe damage to economically important crops through feeding and oviposition. Thrips are prevalent in soybean fields worldwide [1,2,4,5]. Due to their small size and cryptic lifecycle stages, they are often difficult to detect and were previously considered minor pests in soybean fields [1,5,6]. However, their rapid reproductive rates and ability to produce multiple generations per year contribute to increasing population densities, leading to reductions in soybean yield and quality [7]. As a result, thrips have gradually become a major factor restricting the high-quality and high-yield production of soybeans, with infestations becoming increasingly widespread and economically damaging. In the United States, the common thrips species in soybean fields include *Neohydatothrips variabilis* (Beach), *Frankliniella tritici* (Fitch), and *F. fusca* (Hinds). These species not only directly harm soybeans but also spread soybean vein necrosis virus (SVNV), severely affecting soybean quality [8,9]. In Brazil, *F. schultzei* (Trybom) and *Caliothrips phaseoli* (Hood) are the main species in some soybean fields. The population density of *F. schultzei* remains relatively stable across different phenological periods in the northern part of the country [5,6]. Both species can cause a more than 15% reduction in soybean production [10]. In addition, *Frankliniella occidentalis* (Pergande) has been identified as an important pest of vegetables, beans, and other crops in Asia, America, and Africa, causing severe economic losses [11,12].

In China, the damage caused by pest thrips to soybean crops has been frequently reported. For example, *Megalurothrips usitatus* (Bagnall) caused damage to soybean in Taiwan Province; *Thrips nigropilosus* Uzel has severely affected soybean fields in Keshan Farm, Heilongjiang Province; and *Thrips tabaci* Lindeman has infested large areas of Mudanjiang, Jiamusi, and Qiqihar in Heilongjiang Province [1]. With the expansion of soybean cultivation areas and changes in cropping patterns, new pests are constantly emerging in soybean production areas [13], highlighting the need for in-depth research on thrips in soybean fields. Among thrips species, *Thrips flavus* Schrank (Thysanoptera: Thripidae) is broadly distributed worldwide, occurring in Europe and Asia [14,15,16,17,18]. It is recognized as an important pest in agricultural and forestry systems. *T. flavus* can infest a diverse range of host plants, with documented occurrences on species from at least 33 families, including soybean, cotton, citrus, apple, and spruce [15,16,19]. Thrips are considered to have a high invasive potential, given their various dispersal mechanisms [20]. Soybean is one of the most suitable host plants for *T. flavus* [21]. In recent years, this pest has transitioned from a minor issue to a major threat in soybean fields across Northeast China, with increasing impacts on soybean production [1]. Previous studies have investigated the bio-ecology of *T. flavus*. For example, when soybeans are used as a host plant, the lifespan of female thrips is 16.66 days at 22 °C, while males live for 17.0 days at 25 °C [15]. Additionally, CO_2_ levels also influence thrips’ growth and development, with elevated CO_2_ reducing survival while accelerating development [22]. Plant essential oils, particularly those derived from plants in the families Rutaceae and Lamiaceae, have shown toxicity and control efficacy against *T. flavus* [23,24]. For instance, orange leaf oil, zanthoxylum oil, and pomelo peel oil exhibit LC_50_ values of 0.26, 0.27, and 0.44 g/L, respectively [23], while marjoram oil, clary sage oil, and perilla leaf oil have LC_50_ values of 0.41, 0.42, and 0.43 mg/mL, respectively [24]. Although these plant essential oils present a potential alternative to insecticides, their control efficacy is substantially lower during acute pest control situations.

Previously, several insecticides have been evaluated for their use in controlling *T. flavus*. Avermectin and thiamethoxam have demonstrated strong toxicity and efficacy against *T. flavus*, making them promising candidates for pest control [25]. Sun et al. (2024) examined the susceptibility of *T. flavus* in Yunnan Province to imidacloprid, avermectin, and lambda-cyhalothrin over multiple years [26]. They also determined the impact of imidacloprid on thrips population growth through cage tests [26]. Thiamethoxam and imidacloprid are both neonicotinoid insecticides. Currently, about 30% of the insecticides used globally are neonicotinoids [27]. Abamectin, a biogenic insecticide, exhibits significant insecticidal toxicity to pests [28]. Lambda-cyhalothrin, a widely used pyrethroid insecticide in China, has become less effective due to the development of pest resistance resulting from long-term use [29]. The evaluation of the effectiveness of these insecticides against thrips is of value. However, the limited number of insecticides studied so far is insufficient to meet the control needs for soybean pests.

Currently, no insecticide products are specifically registered for controlling thrips infesting soybeans in China. To address this gap, this study aimed to evaluate the toxicity and efficacy of thirty insecticides against *T. flavus*. Laboratory bioassays were first conducted to assess the toxicity of these insecticides against *T. flavus.* Pot experiments were then conducted to evaluate their insecticide efficacy, and the most effective insecticides were selected for subsequent field trials. The results identified several insecticides with strong potential against *T. flavus*. These findings provide a valuable reference for selecting appropriate insecticides for *T. flavus* control in soybean fields and offer a foundation for expanding the registration of insecticides for this purpose in China.

## 2. Materials and Methods

### 2.1. Insect

*Thrips flavus* adults were collected from the soybean field of the Ministry of Agriculture and Rural Development (Jilin) Innovation Center of Soybean Region Technology (43°48′28″ N, 125°25′17″ E, Changchun, China). Notably, the test site was not sprayed with any insecticide prior to collection. Adult thrips were initially captured using the sweeping net method, followed by suction trapping. The specimens were brought to the laboratory and then identified under a stereomicroscope to confirm their identity as *T. flavus*. The thrips were fed soybean leaves (soybean variety ‘Jinong 28’) in a constant temperature incubator (GXZ380B, Jiangnan Instrument Factory, Ningbo, China) at 25 ± 1 °C, with 70% ± 5% relative humidity (RH) and a 16 h:8 h photoperiod for more than one generation [21].

### 2.2. Insecticides

These insecticides were selected based on their registration status in China and their mechanism of action. All insecticides used were registered to control various thrips species on crops other than soybeans. Basic information on the thirty chemical insecticides is shown in Table S1. The hazard classification of each insecticide was determined according to the guidelines of the World Health Organization [30]. The mode of action and classification of the selected insecticides were referenced from the Insecticide Resistance Action Committee (IRAC) (https://irac-online.org/) (8 April 2025).

### 2.3. Method of Laboratory Bioassay

**Figure 1:**
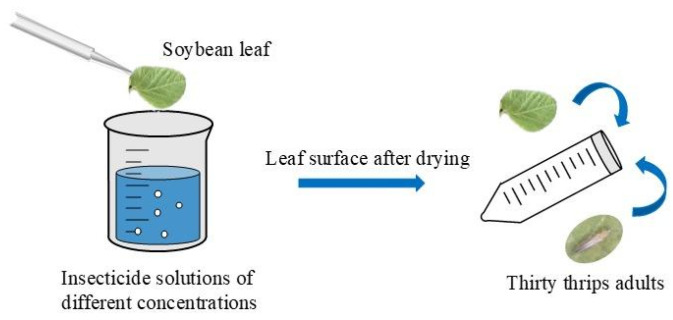
Schematic diagram of the laboratory bioassay.

The method for determining the toxicity of insecticides against *T. flavus* was previously described by Pei et al. [31]. Soybean leaves (variety ‘Jinong 28’) of approximately uniform size (5.0 × 2.5 cm) were rinsed with distilled water and air-dried for about 20 min. Test solutions were prepared by diluting the insecticides with distilled water to the required concentrations. A pre-experimentation was performed to determine the optimal concentration gradient for establishing a probit model before the formal test. The insecticide dose range that induces 16% to 84% mortality in the test population was determined by initially assessing the mortality of the test insects at three to four widely varying doses. The dose gradient was then adjusted within this range, moving up and down the dose gradient to ensure that accurate results were obtained [32]. Each insecticide was diluted to obtain five different concentrations (Table S2). Soybean leaves were submerged in the solution for 10 s and then air-dried at room temperature. Next, thirty *T. flavus* adults were introduced into 50-mL plastic centrifuge tubes through the centrifuge port. The opening of each tube was sealed with parafilm sealing film, which was punctured with approximately 70 micro-holes to allow ventilation (Figure 1). The treated thrips were maintained in controlled conditions (25 ± 1 °C, 70% ± 5% RH, 16 h:8 h photoperiod). For each insecticide concentration combination and control, 30 female adults (2-day-old) were used, and three replicates were performed. To confirm the identity of females, individual thrips were examined under a stereomicroscope (SZ61, Olympus Corporation, Tokyo, Japan) to observe the distinct ovipositor at the end of the abdomen [15]. Mortality was recorded after 24 h.

The mortality rate, adjusted mortality ratio, and relative bioactivity index were calculated according to Equations (1), (2), and (3), respectively [25].

(1) $$M_{1}=\frac{N_{1}}{N}\times 100$$

(2) $$M_{2}=\frac{T_{2}-T_{1}}{1-T_{1}}\times 100$$

(3) $$R=\frac{V_{2}}{V_{1}}\times 100$$

Here, *M*_1_ is the mortality rate; *N* is the total number of insects treated; *N*_1_ is the number of dead insects; *M*_2_ is the adjusted mortality ratio; *T*_1_ is the blank control mortality rate; *T*_2_ is the treatment mortality rate; *R* is the relative bioactivity index; *V*_1_ is the LC_50_ value of the test insecticide; *V*_2_ is the LC_50_ value of the standard insecticide. The insecticide with the greatest LC_50_ value is used as the standard insecticide in the calculation, and its relative bioactivity index is defined as 1.

### 2.4. Method of Pot Experiment

The insecticide efficacy of thirty insecticides against *T. flavus* was evaluated following the method described by Pei et al. [31], with slight modifications. For the pot experiments, soybean plants (variety ‘Jinong 28’) were planted in plastic pots with an outer diameter of 13.5 cm and a height of 10 cm. Each pot was filled with about 700 g of soil and supplemented with 4 g of sulfur-coated slow-release fertilizer (Anshan Sinuo Chemical Co., Ltd., Anshan, China). Nine soybean seeds were sown per pot, and the plants were irrigated and maintained under greenhouse conditions until the seedlings developed a second pair of true leaves (about 15 days). Only one soybean seedling was retained in each pot to ensure healthy growth. The stem of each seedling was surrounded with a cardboard shell plate to prevent dead thrips from falling onto the soil. No additional watering was performed throughout the experiment. Based on the results of the laboratory bioassay, five concentrations were set for each insecticide and prepared in distilled water according to the pre-determined concentration gradients (Table S3). Dilutions were standardized to simulate a field application rate of 900 L of water per 1 hm^2^ of soybean field. For each concentration, 100 mL of diluted solution was prepared. A 5 mL aliquot of the diluted insecticide solution was applied to each pot using a hand sprayer. The control group was sprayed with distilled water. Once the spray solution had completely dried, each pot was covered with a mesh gauze, and thirty adult thrips were introduced. Thrips were sucked from the insect cage with a suction device into centrifuge tubes and subsequently released from the centrifuge tube onto the potted soybean plants. For each insecticide concentration and the control, 30 female adults were used, and three replicates were performed. The treated pots were arranged 1 m apart in a randomized block design under greenhouse conditions. Mortality was recorded at 1, 3, and 7 days after spraying. The insecticide efficacy was calculated using Equation (4) [25].

(4) $$IE=\left(1-\frac{T_{2}\times C_{1}}{C_{2}\times T_{1}}\right)\times 100$$

where: *IE* is the insecticide efficacy, *T*_1_ is the number of thrips in the treatment group before treatment with insecticides, *T*_2_ is the number of thrips in the treatment group after treatment with insecticides, *C*_1_ is the number of thrips in the control group before treatment, and *C*_2_ is the number of thrips in the control group after treatment.

### 2.5. Field Experiment

A field experiment was conducted from 31 July 2023 to 22 August 2023 at the soybean experimental field of Jilin Agricultural University Teaching and Research Base (43°49′16″ N, 125°23′52″ E). Plots measuring 20 m^2^ were established using the soybean variety ‘Jiyu 47’. Plants were arranged in rows spaced 60 cm, with 15 cm between individual plants and 1 m between plots. The total sowing density was 15,000 plants/667 m^2^ (Figure S1). The plots were arranged in a randomized block design, and standard agronomic practices were followed, excluding pesticide application. Four insecticides, imidacloprid, fenthion, sulfoxaflor, and cyetpyrafen, were selected for field evaluation based on their high toxicity and efficacy observed in laboratory and pot experiments. No additional pesticides were applied throughout the soybean growing season. Five concentrations were tested for each of the four insecticides, as follows: (1) cyetpyrafen at 0.90, 2.70, 4.51, 6.29, and 8.10 g a.i.·hm^−2^; (2) imidacloprid at 2.25, 4.50, 6.75, 9.00, and 11.25 g a.i.·hm^−2^; (3) sulfoxaflor 0.40, 0.59, 0.79, 0.99, and 1.19 g a.i.·hm^−2^; and (4) fenthion at 2.25, 4.50, 6.75, 9.00, and 11.25 g a.i.·hm^−2^. Dilutions were standardized to 900 L water per 1 hm^2^ of soybean field to which the solution was applied. Treatments were applied using a Delixi^®^ hand sprayer (Delixi Group Co., Ltd., Shanghai, China) at 0.2–0.4 MPa, equipped with a hand-held wand sprayer with whirlpool nozzles without filters. Tap water was used for the control group. To evaluate treatment efficacy, 20 plants were randomly selected per plot. Each plant was gently shaken over a plastic disc placed beneath it, and the number of thrips dislodged onto the disc was recorded. The number of thrips per plant was determined one day before application and 1, 3, 7, and 14 days after application. Each insecticide concentration and the control were tested in triplicate. The population decline rate and field efficacy were calculated using Equations (5) and (6), respectively [33].

(5) $$P=\frac{P_{0}-P_{1}}{P_{0}}\times 100$$

(6) $$FE=\frac{PR_{1}-PR_{0}}{1-PR_{0}}\times 100$$

where: *P* is the population decline rate; *P*_0_ is the number of individual pests before application; *P*_1_ is the number of individual pests after application; *FE* is field efficacy; *PR*_0_ is the population decline rate in the control area; *PR*_1_ is population decline rate in the threat area.

### 2.6. Data Analysis

Mortality data were subjected to probit analysis to determine the log-dose response, slopes, 95% confidence intervals, and the median lethal concentration (LC_50_) expressed in mg L^−1^. Mortality rates were corrected using values obtained from the control. All analyses were performed using DPS 20.05 software (Hangzhou Ruifeng Information Technology Co., Ltd., Hangzhou, China) [34]. Additionally, a one-way analysis of variance (ANOVA) was conducted using IBM SPSS Statistics version 26.0 (International Business Machines Corporation, Armonk, NY, USA), followed by Tukey’s post hoc test (*p* < 0.05) to determine significant differences in means among treatments. Graphical representations of the data were generated using GraphPad Prism 9.50 (GraphPad Software, Boston, MA, USA).

## 3. Results

### 3.1. Laboratory Bioassay

In the laboratory bioassay, a lower LC_50_ value corresponded to a higher relative bioactivity index of the insecticide, indicating greater toxicity. The LC_50_ values were estimated using the Probit Model, with the correlation coefficient used to assess the goodness-of-fit. A higher correlation coefficient suggests a better fit for the model. The chi-square test was employed to assess the model’s reliability; a lower chi-square value reflected a smaller discrepancy between the observed and expected values. Two insecticides were considered significantly different if their 95% confidence intervals did not overlap. Table 1 presents the observed toxicity of thirty insecticides against *T. flavus*. Among the tested insecticides, fenthion exhibited the highest toxicity against *T. flavus*, with an LC_50_ value of 2.26 mg/L, followed by sulfoxaflor and cyetpyrafen, with LC_50_ values of 4.28 mg/L and 4.94 mg/L, respectively. In contrast, pyriproxyfen, cyflumetofen, monosultap, and thiacloprid showed relatively low toxicity, with LC_50_ values of 63.72, 67.26, 88.34, and 144.77 mg/L. Among neonicotinoid insecticides, sulfoxaflor demonstrated the highest toxicity, while thiacloprid was the least toxic. The LC_50_ values of neonicotinoids ranged from 4.28 to 144.77 mg/L. Among pyrethroid insecticides, fenpropathrin exhibited the highest toxicity, whereas beta-cypermethrin showed the lowest. Among diamide insecticides, chlorantraniliprole had the highest toxicity, with an LC_50_ value of 13.78 mg/L. Among the organophosphates, fenthion was significantly more toxic than malathion. For the acaricides insecticides, cyflumetofen had the lowest toxicity.

### 3.2. Pot Experiment

The pot experiment indicated that the insecticidal efficacy of different insecticides against *T. flavus* varied significantly over time and with increasing application concentrations (Table 2). Seven days after application, sulfoxaflor, cyetpyrafen, chlorantraniliprole, fenthion, imidacloprid, and bifenazate were the most effective insecticides. Specifically, cyetpyrafen achieved an efficacy of 98.81% ± 1.19%, while sulfoxaflor, fenthion, chlorantraniliprole, bifenazate, and malathion reached 100%.

The efficacies of the thirty insecticides against thrips at their maximum concentrations are shown in Table S4. One day after application, bifenazate exhibited significantly higher efficacy than all other insecticides except for sulfoxaflor (*F* = 14.482, *df* = 29, *p* < 0.001). At three days post-application, no significant differences in efficacy were observed among bifenazate, sulfoxaflor, cyetpyrafen, malathion, chlorantraniliprole, chlorfenapyr, and pyridaben; however, bifenazate was significantly more effective than the remaining insecticides (*F* = 11.611, *df* = 29, *p* < 0.001). Seven days after application, significant differences were observed among the thirty insecticides. No significant differences were observed among fenthion, sulfoxaflor, cyetpyrafen, imidacloprid, bifenazate, malathion, and chlorantraniliprole (*F* = 10.517, *df* = 29, *p* < 0.001).

### 3.3. Field Efficacy Experiment

The field efficacy of imidacloprid, fenthion sulfoxaflor, and cyetpyrafen against *T. flavus* followed a trend of increasing efficacy that peaked seven days after application, followed by a subsequent decline (Table 3). Peak efficacies recorded on day 7 were as follows: imidacloprid (11.25 g a.i.·hm^−2^) at 82.91% ± 3.63%, fenthion (11.25 g a.i.·hm^−2^) at 84.23% ± 2.05%, sulfoxaflor (.19 g a.i.·hm^−2^) at 80.23% ± 2.08%, and cyetpyrafen (8.10 g a.i.·hm^−2^) at 85.68% ± 0.44%. No significant differences in efficacy were observed among the four insecticides at different concentrations on day 1 (*F* = 1.48, *df* =19, *p* = 0.146), day 3 (*F* = 1.81, *df* =19, *p* = 0.057), and day 14 (*F* = 1.01, *df* =19, *p* = 0.475) after application. However, on day 7, the efficacy of imidacloprid (11.25 g a.i.·hm^−2^), fenthion (11.25 g a.i.·hm^−2^), and cyetpyrafen (8.10 g a.i.·hm^−2^) was significantly higher than that of cyetpyrafen at 2.70 g a.i.·hm^−2^ (*F* = 2.63, *df* =19, *p* = 0.005).

## 4. Discussion

The objective of this study was to identify effective insecticides against *T. flavus* and to lay the foundation for its integrated management. The toxicity, insecticide efficacy, and field performance of thirty insecticides against *T. flavus* were evaluated individually. Results from the laboratory bioassay indicated that fenthion, sulfoxaflor, cyetpyrafen, and imidacloprid exhibited relatively high toxicity against *T. flavus*. Although the efficacies of cyetpyrafen and imidacloprid did not rank among the highest, they were not significantly different from those of the five insecticides that achieved 100% control seven days after application. A comparison of LC_90_ values showed that fenthion, sulfoxaflor, cyetpyrafen, and imidacloprid had lower values, aligning with the results of the insecticidal efficacy. These findings suggest that effective control of *T. flavus* can be achieved with lower doses of these four insecticides. Consequently, imidacloprid, fenthion, sulfoxaflor, and cyetpyrafen were selected for further evaluation in field trials. However, the field efficacy of all four chemicals declined over the 14-day test period, possibly due to rainfall during this period or other environmental factors.

### 4.1. Neonicotinoid Group

Imidacloprid, acetamiprid, dinotefuran, sulfoxaflor, thiacloprid, and nitenpyram are neonicotinoids [27,35], among which imidacloprid and sulfoxaflor were determined as effective against *T. flavus* in the present study. Sun et al. (2024) found that the LC_50_ of *T. flavus* populations treated with imidacloprid in the Heilongtan area of Yunnan Province was 67.63 mg/L, which was lower than LC_50_ observed in the Dabai area (LC_50_ = 96.15 mg/L) [26]. This difference may be attributed to the frequent use of insecticides in the Dabai area, potentially leading to pest resistance [26]. These values were significantly higher than the LC_50_ of imidacloprid against *T. flavus* (LC_50_ = 6.16 mg/L) determined in the present study. The imidacloprid also demonstrated substantial toxicity to *Frankliniella invasor* Sakimura in mango orchards, with an LC_50_ of 5.428 mg/L [36]. Effective suppression of *S. dorsalis* in pepper fields was observed when imidacloprid was applied at 40 g a.i.·hm^−1^ [37]. Numerous studies have explored control strategies for thrips. Shen et al. (2023) investigated the sensitivity of *F. occidentalis* and *T. palmi* Karny populations to sulfoxaflor across different host plants and geographic regions [38]. An *F. occidentalis* population in the Beijing area was found to be more susceptible to sulfoxaflor, with relatively low LC_50_ values ranging from 20.71 to 43.17 mg/L. However, *T. palmi* populations from Hainan, China, showed more tolerance to this compound [38]. In the present study, sulfoxaflor demonstrated the highest toxicity against *T. flavus*, with an LC_50_ value of 4.28 mg/L. Its efficacy in pot and field experiments reached 100% and 80.23%, respectively, at a dosage of 1.19 g a.i.·hm^−2^, seven days after application. The field efficacy results of this insecticide were better than those previously reported for *Scirtothrips dorsalis* Hood [39] and comparable to the results reported by Renkema et al. (2018) for flower thrips in strawberry fields [40]. These differences may be attributed to variations in thrips species, host plants, and geographical locations, all of which can influence thrips’ susceptibility to insecticides [6,38,41].

Previous studies have reported that sulfoxaflor is toxic to *Amblyseius swirskii* Athias-Henriot (Acari: Phytoseiidae), a natural predator of *F*. *occidentalis*. Under laboratory conditions, the mortality rate of *A*. *swirskii* increased by 19.53% after treatment with sulfoxaflor at 60 a.i. mg/L compared to the untreated group [42]. Thus, the potential risks that neonicotinoid insecticides present to natural predators and pollinators cannot be overlooked. Neonicotinoid insecticides exhibit systemic activity, and natural enemy insects feeding on plants or prey contaminated with the active ingredient can experience proportional effects [43]. Neonicotinoid seed coating leads to a reduced ability of honey bee species to establish new populations in the year following exposure [44]. Therefore, it is crucial to consider the potential effects of neonicotinoid insecticides on non-target insects when implementing their use.

The target site of neonicotinoid insecticides is the nicotinic acetylcholine receptor (nAChR). These insecticides function as potent nAChR agonists, mainly by circulating in the central nervous system. This can disrupt the neurotransmission process involving choline, ultimately leading to insect death [45]. *Gynaikothrips uzeli* (Zimmermann) adults and nymphs treated with imidacloprid showed significantly lower phenoloxidase activity compared to the control [46]. In the field, when *Frankliniella occidentalis* and *F. intonsa* (Trybom) were exposed to imidacloprid, the activities of carboxylesterase, acetylcholinesterase, mixed function oxidase, catalase, and peroxidase were significantly increased, while the superoxide dismutase activity was significantly decreased [47]. The resistance of *T. palmi* to imidacloprid is associated with CYP450-mediated detoxification of the nicotinic acetylcholine receptor β1 subunit determined by amino acid position 81, which encodes the susceptible amino acid arginine [48]. Previous research has identified two segregating mutations in the β1 subunit of nAChR, V65I, and V104I. The V65I mutation significantly reduces the binding ability of nAChR to sulfoxaflor, while the V104I mutation decreases agonist affinity, thereby reducing the binding ability of nAChR to sulfoxaflor [49]. These studies may provide a reference for future insecticide research, particularly concerning the mode of action and resistance mechanisms in thrips.

### 4.2. Pyrethroid and Diamide Group

In this study, fenpropathrin exhibited greater toxicity and insecticide efficacy than beta-cypermethrin and lambda-cyhalothrin. Although fenpropathrin displayed relatively low toxicity to western flower thrips, its efficiency was moderately enhanced when combined with acephate [50]. It is worth noting that, among the thirty insecticides tested, lambda-cyhalothrin, which demonstrated high toxicity in the present study, showed relatively low toxicity to *T. flavus* in the Dabai area of Yunnan Province in 2019 (LC_50_ = 215.45 mg/L) [26]. Tetraniliprole showed substantial toxicity (LC_50_ = 22.35 mg/L) in the laboratory. However, its performance in the subsequent pot experiment was suboptimal, with an efficacy of 67.12% ± 9.49% at an application rate of 18.00 g a.i.·hm^−2^ seven days after application. Previous studies have reported significant variations in viscosity, surface tension, and droplet size among different dosage forms [51]. In the present laboratory tests, *T. flavus* exhibited limited mobility. However, in the pot experiment, their mobility was greater, which might have reduced their contact with the insecticide. These factors may account for the discrepancies observed between the test results. Using a combination of air-assisted and electrostatic systems can improve spray deposition, droplet coverage, and density on soybean leaves [52], which is a promising approach to enhance insecticide efficacy.

### 4.3. Organophosphate and Nereistoxin Group

Fenthion and malathion are organophosphorus insecticides [53,54], a class widely used because of their ability to inactivate the enzyme acetylcholinesterase [55]. The LC_50_ values of malathion against western flower thrips populations on blackberries in various regions of Mexico ranged from 3.96 to 274.80 mg/L [56]. Although organophosphorus insecticides are usually considered highly toxic compounds, fenthion and malathion are less toxic and are still employed for pest control in China. Nevertheless, organophosphorus residues in agricultural products and the environment have been of widespread concern [57]. While fenthion demonstrated good control efficacy against *T. flavus*, its continued use may be phased out in the future. Moreover, few studies have focused on its effectiveness against thrips. In the present study, three nereistoxin insecticides exhibited relatively poor efficacy against *T. flavus*.

### 4.4. Acaricide Group

The insecticides cyflumetofen, cyenopyrafen, and cyetpyrafen tested in the present study belong to the group of mitochondrial electron transport complex II (METI-II) inhibitors, which are newly developed and commercialized acaricides [58,59,60,61]. Cyetpyrafen showed the highest contact activity against *Thrips hawaiiensis* (Morgan), with a lethal median rate (LR_50_) of 7.49 g a.i.·ha^−1^ 24 h after treatment, which was much lower than the recommended field dosage. Moreover, its use does not negatively impact the natural thrips predator *Orius strigicollis* (Poppius) [62]. Cyetpyrafen requires a series of metabolic transformations in the pest’s body, such as decarboxylation, and hydrolysis, to form its active form, which exerts toxic effects on the pest [63]. Both cyetpyrafen and its metabolites may be toxic to aquatic organisms; therefore, their ecotoxicity should be considered in future studies [64]. In the present study, the toxicity of cyetpyrafen against *T. flavus* was measured in the laboratory bioassay (4.94 mg/L at 24 h after application), consistent with the above finding. The field efficacy of insecticides is influenced not only by the applied concentration but also by factors such as the persistence of the insecticide and, importantly, the history of insecticide application in each soybean-producing region [65]. This study also included several acaricide products because some are registered for thrips control in China. Therefore, we tested the insecticidal activity of the acaricides against *T. flavus*.

The results of this study also suggest a notable phenomenon: insecticides belonging to the same chemical group may exhibit substantial differences in toxicity and control efficacy against *T. flavus*. From the perspective of insect target sites, these variations may be attributed to differences in the binding affinity of insecticides to the receptor. For example, the structural diversity of nAChR subunits contributes to differences in the binding capacity of neonicotinoid insecticides [66]. Amino acid mutations at insecticide target sites can also contribute to differences in insecticide toxicity [67]. In addition, insects may upregulate the expression of detoxification enzymes in response to insecticide pressure, and the efficiency of detoxification pathways varies depending on the chemical structure of the compounds [68]. From the perspective of insecticide formulations, variations in the active ingredients of the products and the presence of specific additives may also influence efficacy. Furthermore, the toxicological effects of insecticides are closely linked to their ability to penetrate insect cuticles [69], and differences in cuticular permeability may lead to variations in insecticide performance across compounds.

In soybean production areas of Northeast China, where flower and leaf damage are closely associated with yield loss, thrips are the main pest with rasping mouthparts [1]. Prolonged insecticide application can lead to the development of resistance in thrips populations [70]. Although no resistance cases have been reported in this region, the potential risk cannot be ignored. This study evaluated cyetpyrafen fenthion, sulfoxaflor, and imidacloprid for their control efficacy. Among them, cyetpyrafen and sulfoxaflor are associated with slightly lower control costs compared to imidacloprid and fenthion. Currently, imidacloprid is widely used as the active ingredient in insecticide products for aphid control in China’s soybean fields, whereas sulfoxaflor has not yet been registered for use. Although this study clarified the insecticidal effectiveness of sulfoxaflor against *T. flavus*, the results alone are insufficient to support its registration in any specific region or country. Prior to incorporation into integrated pest management strategies, further trials are required to assess their impact on natural enemies, pollinators, and the environment to ensure their safety [42,43,44,57,62]. Fenthion, sulfoxaflor, and imidacloprid are classified as Class II (moderately hazardous) by the WHO, and their application should be carefully managed to minimize risk to non-target organisms [30].

It is important to note that, in addition to the four insecticides selected for field trials, several other insecticides have demonstrated high toxicity and persistence. Agents such as chlorantraniliprole, which exhibit high toxicity and efficacy, can serve as effective alternatives to mitigate the development of resistance. *T. flavus* was mainly found in the upper leaves of soybean plants from the first to the fifth leaf position, with significantly higher numbers of adults and nymphs on the abaxial leaf surface than on the adaxial surface. Therefore, these areas should be prioritized when spraying. Spray drift, a prevalent issue during insecticide application, can significantly diminish the efficacy of pest control measures [71]. Preventive measures should be diligently implemented to mitigate this issue. When surveying field populations of thrips, sticky traps are recommended, as they can effectively capture thrips and prevent escape. Combined toxicity and insecticide efficacy results show that insecticides achieving more than 90% efficacy are promising for *T. flavus* management. In addition to controlling thrips, it is crucial to implement appropriate strategies to monitor insecticide resistance in this species in the future [38,41]. Although the risk of resistance due to continuous pesticide application was not assessed in the present trial, the long-term effects could be investigated in future studies through laboratory-based resistance selection experiments. Future research should also prioritize exploring and developing entomopathogenic fungi and plant essential oils as eco-friendly insecticide alternatives to mitigate dependence on chemical insecticides, enhance thrips control efficacy, and delay the development of pest resistance [72,73].

## 5. Conclusions

Among the insecticides subjected to laboratory tests, fenthion, sulfoxaflor, cyetpyrafen, and imidacloprid exhibited the highest toxicity against *T. flavus*. The insecticide efficacy of sulfoxaflor, chlorantraniliprole, and bifenazate reached 100%. In the field, the highest concentrations of fenthion, sulfoxaflor, cyetpyrafen, and imidacloprid demonstrated efficacy exceeding 80%. Overall, sulfoxaflor and cyetpyrafen are promising insecticides for future applications in the integrated management of *T. flavus*. Insecticides with efficacy exceeding 90% show potential for effective control of *T. flavus* and should be considered for expanded registration to enable their use in soybeans for thrips control.

## Figures and Tables

**Table 1:** Toxicity of thirty insecticides against *Thrips flavus* (Changchun, Jilin Province, 2023).

Group	Insecticide	Probit Model	Correlation Coefficient	LC50 (mg/L) 95% Confidence Interval	LC_9_0 (mg/L) 95% Confidence Interval	Relative Bioactivity Index	Chi-Square
Neonicotinoids	Sulfoxaflor	y = 2.19 + 4.45x	0.96	4.28 3.68–5.56	8.30 6.14~20.78	33.86	1.26
	Imidacloprid	y = 3.13 + 2.74x	0.85	6.16 0.82–11.10	21.32 12.50~29.85	23.52	2.28
	Nitenpyram	y = 1.92 + 2.20x	0.82	24.91 15.24–32.47	95.15 69.62~181.38	5.81	7.11
	Dinotefuran	y = −6.31 + 7.17x	0.97	37.69 25.45–41.20	56.87 50.60~101.67	3.84	0.43
	Acetamiprid	y = 0.97 + 2.37x	0.96	50.18 36.69–66.70	174.24 109.51~690.93	2.89	1.55
	Thiacloprid	y = 10.96 + 7.39x	0.91	144.77 129.81–158.36	215.86 185.93~346.61	1.00	3.03
Pyrethroids	Fenpropathrin	y = 0.93 + 3.63x	0.96	13.20 2.75–16.81	29.76 24.80~85.24	10.96	0.67
	lambda-Cyhalothrin	y = 2.65 + 1.89x	0.88	19.67 7.20–28.65	99.63 67.72~284.07	7.36	3.88
	beta-Cypermethrin	y = −1.02 + 3.46x	0.95	55.25 44.02–65.17	129.74 99.07~251.80	2.62	2.06
Diamides	Chlorantraniliprole	y = 2.88 + 1.86x	0.86	13.78 8.62–18.36	67.20 39.80~326.28	10.51	4.54
	Tetrachlorantraniliprole	y = 1.18 + 3.34x	0.99	13.92 10.31–16.90	33.68 27.77~45.41	10.40	1.12
	Cyantraniliprole	y = 2.30 + 2.22x	0.89	16.33 9.18–21.62	61.60 42.93~152.38	8.87	4.20
	Tetraniliprole	y = 1.91 + 2.29x	0.93	22.35 10.24–31.53	81.11 61.93~133.47	6.48	3.28
Nereistoxins	Cartap	y = −0.72 + 3.25x	0.98	57.25 33.23–69.32	141.87 109.69~341.79	2.53	2.12
	Bisultap	y = −1.51 + 3.88x	0.96	47.60 38.40–56.67	101.79 79.42~175.91	3.04	3.18
	Monosultap	y = −2.63 + 3.92x	0.99	88.34 75.88–113.24	187.48 134.67~571.45	1.64	0.45
Organophosphates	Fenthion	y = 4.20 + 2.27x	0.86	2.26 0.19–4.68	8.31 3.29~11.86	64.08	1.65
	Malathion	y = 2.20 + 2.53x	0.98	12.73 6.81–16.84	40.86 31.09~74.56	11.37	1.97
Acaricides	Cyetpyrafen	y = 3.90 + 1.58x	0.88	4.94 2.09–7.09	31.87 21.37~71.07	29.29	4.42
	Cyenopyrafen	y = 3.13 + 1.69x	0.86	12.62 3.45–18.83	73.21 45.08~426.62	11.48	3.39
	Chlorfenapyr	y = 1.74 + 2.85x	0.80	13.99 8.20–18.34	39.46 31.06~59.78	10.35	3.36
	Spirotetramat	y = 2.38 + 2.09x	0.91	17.97 12.01–23.88	73.71 44.97~330.43	8.06	3.92
	Pyridaben	y = 0.65 + 3.20x	0.94	22.75 18.16–26.96	57.18 45.72~83.82	6.36	5.53
	Bifenazate	y = −0.63 + 3.51x	0.96	40.23 31.66–47.19	93.26 76.25~134.34	3.59	2.94
	Spirodiclofen	y = −3.46 + 4.77x	0.96	59.65 51.67–67.88	110.80 91.21~165.43	2.43	2.8
	Cyflumetofen	y = −10.88 + 8.69x	0.95	67.26 59.53–72.20	94.47 87.66~107.96	2.15	2.33
Others	Buprofezin	y = −1.25 + 3.97x	0.97	37.64 30.66–44.10	79.23 60.54~180.90	3.85	1.30
	Flonicamid	y = −4.89 + 5.81x	0.99	50.62 36.17–57.00	84.19 74.61~118.95	2.86	0.29
	Pymetrozine	y = 0.78 + 2.78x	0.74	50.43 29.25–66.24	165.79 123.63~309.02	2.87	5.75
	Pyriproxyfen	y = −12.51 + 9.71x	0.98	63.72 59.49–69.80	86.36 76.60~112.75	2.27	0.86

**Table 2:** Insecticide efficacy of thirty insecticides against *Thrips flavus* in pot experiment (Changchun, Jilin Province, 2023).

Group	Insecticides	Dose (g a.i.·hm^−2^)	Insecticide Efficacy (%)		
			Days After Application (day)		
			1	3	7
Neonicotinoids	Sulfoxaflor	0.40	33.74 ± 7.90 ^b^	41.03 ± 6.79 ^c^	60.27 ± 1.37 ^d^
		0.59	45.78 ± 4.17 ^b^	53.85 ± 5.87 ^bc^	72.60 ± 3.62 ^cd^
		0.79	51.81 ± 3.19 ^b^	62.82 ± 6.78 ^bc^	78.08 ± 3.62 ^bc^
		0.99	67.47 ± 2.08 ^ab^	79.49 ± 3.39 ^b^	87.67 ± 2.37 ^b^
		1.19	89.16 ± 5.52 ^a^	97.44 ± 2.56 ^a^	100.00± 0 ^a^
	Imidacloprid	2.25	23.81 ± 3.15 ^a^	30.12 ± 4.34 ^a^	51.90 ± 6.70 ^b^
		4.50	28.57 ± 4.12 ^a^	42.17 ± 3.61 ^a^	72.15 ± 2.53 ^b^
		6.75	28.57 ± 3.57 ^a^	42.17 ± 6.26 ^a^	72.15 ± 5.52 ^b^
		9.00	29.76 ± 6.30 ^a^	45.78 ± 4.17 ^a^	93.67 ± 3.35 ^a^
		11.25	38.10 ± 3.15 ^a^	53.01 ± 5.52 ^a^	98.73 ± 1.27 ^a^
	Nitenpyram	2.88	40.96 ± 6.71 ^a^	47.44 ± 6.78 ^ab^	54.79 ± 2.37 ^c^
		5.94	31.32 ± 9.56 ^a^	44.87 ± 5.59 ^ab^	75.34 ± 6.28 ^bc^
		9.00	31.32 ± 5.52 ^a^	39.74 ± 10.01 ^b^	43.84 ± 8.33 ^ab^
		11.88	49.40 ± 2.09 ^a^	58.97 ± 4.62 ^ab^	61.64 ± 5.97 ^abc^
		14.94	59.04 ± 1.20 ^a^	73.08 ± 4.44 ^a^	83.56 ± 2.37 ^a^
	Dinotefuran	6.84	4.76 ± 1.19 ^c^	14.46 ± 2.41 ^b^	31.65 ± 5.80 ^b^
		7.38	5.95 ± 1.19 ^c^	28.92 ± 3.19 ^ab^	40.51 ± 5.52 ^b^
		8.10	10.71 ± 4.13 ^bc^	36.14 ± 4.34 ^ab^	56.96 ± 4.56 ^b^
		8.64	25.00 ± 5.45 ^ab^	50.60 ± 8.69 ^a^	58.23 ± 6.58 ^b^
		9.18	46.43 ± 5.46 ^a^	59.04 ± 10.50 ^a^	93.67 ± 3.35 ^a^
	Acetamiprid	0.90	42.17 ± 2.09 ^b^	52.57 ± 5.59 ^c^	67.78 ± 2.94 ^c^
		1.80	46.99 ± 4.35 ^b^	50.00 ± 4.44 ^c^	66.67 ± 1.93 ^c^
		2.70	51.81 ± 5.25 ^ab^	61.54 ± 2.22 ^bc^	88.89 ± 2.94 ^ab^
		3.60	56.63 ± 5.52 ^ab^	71.79 ± 4.62 ^ab^	77.78 ± 4.84 ^bc^
		4.50	67.47 ± 2.08 ^a^	83.34 ± 1.28 ^a^	94.44 ± 1.11 ^a^
	Thiacloprid	41.98	24.10 ± 5.52 ^b^	36.14 ± 7.90 ^a^	31.51 ± 6.85 ^b^
		47.99	37.35 ± 1.20 ^ab^	53.01 ± 2.09 ^a^	53.42 ± 3.62 ^ab^
		54.00	37.35 ± 3.19 ^ab^	44.58 ± 1.20 ^a^	46.58 ± 4.11 ^ab^
		59.98	46.99 ± 8.43 ^ab^	54.22 ± 7.90 ^a^	53.42 ± 5.48 ^ab^
		65.99	55.42 ± 4.82 ^a^	62.65 ± 4.34 ^a^	69.86 ± 4.94 ^a^
Pyrethroids	beta-Cypermethrin	1.35	8.43 ± 3.19 ^b^	30.77 ± 7.69 ^a^	61.64 ± 2.74 ^b^
		2.03	20.48 ± 7.52 ^ab^	28.21 ± 3.39 ^a^	61.64 ± 8.33 ^ab^
		2.70	28.92 ± 2.41 ^ab^	39.74 ± 2.56 ^a^	76.71 ± 8.33 ^ab^
		3.37	38.55 ± 2.09 ^ab^	58.97 ± 7.14 ^a^	90.41 ± 3.62 ^ab^
		4.04	30.12 ± 8.69 ^a^	51.1 ± 10.17 ^a^	94.52 ± 2.74 ^a^
	lambda-Cyhalothrin	0.75	15.66 ± 3.19 ^b^	26.92 ± 4.44 ^a^	63.01 ± 11.86 ^a^
		1.50	10.84 ± 4.82 ^b^	25.64 ± 3.39 ^a^	64.38 ± 13.07 ^b^
		2.25	26.51 ± 4.34 ^ab^	30.77 ± 2.22 ^a^	68.49 ± 5.48 ^ab^
		3.00	24.10 ± 5.52 ^ab^	32.05 ± 5.59 ^a^	58.90 ± 4.11 ^ab^
		3.75	40.96 ± 4.34 ^a^	55.13 ± 3.39 ^a^	89.04 ± 5.48 ^a^
	Fenpropathrin	2.88	15.66 ± 2.41 ^d^	37.18 ± 2.56 ^b^	49.32 ± 4.94 ^c^
		3.42	40.96 ± 3.19 ^bc^	55.13 ± 4.62 ^b^	71.23 ± 4.11 ^bc^
		3.96	31.33 ± 7.52 ^cd^	55.13 ± 5.59 ^b^	78.08 ± 3.62 ^bc^
		4.50	54.22 ± 1.21 ^ab^	60.26 ± 6.41 ^ab^	91.78 ± 4.11 ^ab^
		5.04	66.26 ± 3.19 ^a^	78.21 ± 4.62 ^a^	97.26 ± 2.74 ^a^
Diamides	Chlorantraniliprole	1.21	50.59 ± 5.39 ^b^	60.00 ± 6.22 ^b^	80.00 ± 4.24 ^b^
		2.39	65.88 ± 13.57 ^ab^	72.94 ± 11.59 ^ab^	87.06 ± 3.11 ^b^
		3.60	75.29 ± 7.35 ^ab^	80.00 ± 6.22 ^ab^	97.65 ± 1.18 ^a^
		4.81	83.53 ± 4.24 ^ab^	87.06 ± 3.11 ^ab^	100.00 ± 0 ^a^
		5.99	87.06 ± 3.11 ^a^	91.76 ± 3.11 ^a^	100.00± 0 ^a^
	Tetrachlorantraniliprole	0.75	39.76 ± 8.43 ^a^	42.31 ± 8.01 ^a^	52.05 ± 10.96 ^a^
		1.50	24.10 ± 2.09 ^a^	41.03 ± 12.62 ^a^	46.58 ± 12.55 ^a^
		2.25	48.19 ± 5.25 ^a^	53.85 ± 6.66 ^a^	68.49 ± 8.98 ^a^
		3.00	25.30 ± 3.19 ^a^	51.28 ± 8.41 ^a^	69.86 ± 13.90 ^a^
		3.75	42.17 ± 5.52 ^a^	44.87 ± 6.78 ^a^	56.16 ± 9.88 ^a^
	Cyantraniliprole	2.70	16.87 ± 8.35 ^a^	15.38 ± 8.88 ^a^	27.40 ± 8.98 ^ab^
		5.40	8.43 ± 1.21 ^a^	11.54 ± 2.22 ^a^	19.18 ± 5.48 ^b^
		8.10	28.92 ± 4.35 ^a^	30.77 ± 5.88 ^a^	42.47 ± 4.74 ^ab^
		10.80	20.48 ± 5.52 ^a^	20.51 ± 5.59 ^a^	36.99 ± 4.94 ^ab^
		13.50	20.48 ± 10.84 ^a^	23.08 ± 9.68 ^a^	56.16 ± 3.62 ^a^
	Tetraniliprole	3.60	15.66 ± 6.38 ^a^	17.95 ± 8.41 ^a^	27.40 ± 9.59 ^a^
		7.20	33.73 ± 3.19 ^a^	39.74 ± 1.28 ^a^	42.47 ± 2.37 ^a^
		10.80	20.48 ± 10.43 ^a^	24.36 ± 11.39 ^a^	32.88 ± 15.80 ^a^
		14.40	22.89 ± 2.41 ^a^	33.34 ± 1.28 ^a^	61.64 ± 19.76 ^a^
		18.00	42.17 ± 6.26 ^a^	56.41 ± 12.23 ^a^	67.12 ± 9.49 ^a^
Nereistoxins	Cartap	44.10	7.23 ± 2.41 ^c^	19.23 ± 2.22 ^c^	27.40 ± 5.97 ^c^
		58.21	38.55 ± 2.09 ^b^	42.31 ± 3.85 ^b^	42.47 ± 2.37 ^bc^
		73.21	45.78 ± 2.09 ^ab^	44.87 ± 2.56 ^b^	46.58 ± 4.74 ^bc^
		88.20	53.01 ± 4.17 ^ab^	57.69 ± 5.88 ^ab^	60.27 ± 1.37 ^ab^
		102.31	59.04 ± 1.20 ^a^	65.38 ± 2.22 ^a^	71.23 ± 4.75 ^a^
	Bisultap	4.50	12.05 ± 3.19 ^b^	14.10± 3.39 ^b^	38.36 ± 7.12 ^c^
		9.00	16.87 ± 3.61 ^ab^	25.64 ± 5.13 ^ab^	57.53 ± 9.59 ^bc^
		13.50	27.71 ± 5.52 ^ab^	35.90 ± 6.78 ^a^	78.08 ± 10.70 ^ab^
		18.00	21.69 ± 2.41 ^ab^	30.77 ± 2.22 ^ab^	94.52 ± 1.37 ^a^
		22.50	32.53 ± 1.20 ^a^	43.59 ± 5.59 ^a^	82.19 ± 7.63 ^ab^
	Monosultap	40.50	7.23 ± 1.21 ^c^	12.82 ± 3.39 ^d^	17.81 ± 2.37 ^c^
		53.46	19.28 ± 1.20 ^c^	26.93 ± 3.85 ^cd^	26.03 ± 2.37 ^c^
		67.23	36.15 ± 3.19 ^b^	38.46 ± 3.84 ^bc^	41.10 ± 4.94 ^b^
		81.00	43.37 ± 5.25 ^ab^	47.44 ± 3.39 ^ab^	54.79 ± 2.37 ^ab^
		93.96	56.63 ± 5.52 ^ab^	60.26 ± 4.62 ^a^	67.12 ± 2.37 ^a^
Organophosphates	Fenthion	2.25	33.74 ± 5.25 ^b^	42.31 ± 5.87 ^b^	58.90 ± 10.87 ^b^
		4.50	38.55 ± 7.52 ^b^	47.43 ± 5.59 ^b^	50.68 ± 2.37 ^b^
		6.75	46.99 ± 8.43 ^ab^	58.97 ± 10.01 ^ab^	68.49 ± 13.07 ^b^
		9.00	38.56 ± 5.52 ^b^	56.41 ± 3.39 ^b^	82.19 ± 1.37 ^b^
		11.25	71.09 ± 3.61 ^a^	84.62 ± 3.85 ^a^	100.00± 0 a
	Malathion	4.05	40.96 ± 6.71 ^b^	62.82 ± 3.39 ^b^	69.86 ± 4.94 ^b^
		7.29	44.58 ± 1.20 ^b^	73.08 ± 4.44 ^ab^	73.97 ± 2.74 ^b^
		10.53	38.55 ± 4.17 ^b^	64.10 ± 13.01 ^b^	80.82 ± 1.37 ^b^
		13.77	56.63 ± 2.09 ^ab^	88.46 ± 2.22 ^ab^	95.89 ± 2.37 ^a^
		17.01	65.06 ± 4.34 ^a^	94.87 ± 2.56 ^a^	100.00± 0 ^a^
Acaricides	Cyetpyrafen	0.90	23.81 ± 3.15 ^d^	41.67 ± 6.63 ^c^	65.48 ± 1.19 ^c^
		2.70	46.43 ± 2.06 ^c^	54.76 ± 3.15 ^bc^	73.81 ± 5.19 ^bc^
		4.51	51.19 ± 1.19 ^c^	66.67 ± 4.29 ^b^	91.67 ± 4.29 ^ab^
		6.29	69.05 ± 2.38 ^b^	75.00 ± 2.06 ^b^	97.62 ± 2.38 ^a^
		8.10	80.95 ± 2.38 ^a^	91.67 ± 3.15 ^a^	98.81 ± 1.19 ^a^
	Cyenopyrafen	2.70	6.02 ± 2.09 ^b^	11.54 ± 2.22 ^c^	36.99 ± 3.62 ^b^
		5.40	32.53 ± 4.34 ^a^	41.02 ± 7.14 ^bc^	72.60 ± 3.62 ^a^
		8.10	37.35 ± 6.71 ^a^	39.74 ± 7.80 ^bc^	75.34 ± 4.75 ^a^
		10.80	46.99 ± 2.41 ^a^	57.69 ± 3.85 ^ab^	76.71 ± 9.88 ^a^
		13.50	59.03 ± 11.49 ^a^	79.49 ± 10.01 ^a^	90.41 ± 3.62 ^a^
	Chlorfenapyr	0.90	37.35 ± 4.34 ^bc^	56.41 ± 3.39 ^b^	58.90 ± 2.37 ^bc^
		1.80	34.94 ± 6.26 ^c^	47.44 ± 7.14 ^b^	47.95 ± 7.62 ^c^
		2.70	53.01 ± 3.61 ^abc^	57.69 ± 3.85 ^b^	69.86 ± 5.97 ^bc^
		3.60	59.04 ± 6.71 ^ab^	64.10 ± 10.01 ^ab^	76.71 ± 6.85 ^ab^
		4.50	69.88 ± 3.19 ^a^	88.46 ± 2.22 ^a^	92.29 ± 2.43 ^a^
	Spirotetramat	1.61	16.87 ± 5.52 ^b^	25.64 ± 4.62 ^b^	34.25 ± 6.28 ^c^
		3.23	32.53 ± 7.33 ^ab^	34.62 ± 6.66 ^ab^	50.68 ± 2.37 ^bc^
		4.84	59.04 ± 6.71 ^ab^	65.39 ± 5.87 ^a^	86.30 ± 4.94 ^ab^
		6.45	43.37 ± 3.19 ^a^	51.28 ± 3.39 ^ab^	83.56 ± 10.34 ^ab^
		8.06	45.78 ± 4.17 ^a^	60.26 ± 11.18 ^a^	87.67 ± 6.28 ^a^
	Pyridaben	1.50	35.37 ± 1.22 ^c^	70.73 ± 2.11 ^b^	80.49 ± 5.32 ^b^
		3.00	51.22 ± 3.23 ^bc^	74.39 ± 3.66 ^b^	81.71 ± 2.11 ^b^
		4.50	59.76 ± 4.22 ^b^	78.05 ± 2.11 ^b^	84.15 ± 3.23 ^b^
		5.99	64.63 ± 8.54 ^ab^	80.49 ± 1.22 ^ab^	87.80 ± 1.22 ^ab^
		7.49	82.93 ± 3.22 ^a^	90.24 ± 3.23 ^a^	96.34 ± 2.11 ^a^
	Bifenazate	9.68	42.35 ± 16.6 ^b^	41.18 ± 5.88 ^b^	75.29 ± 7.35 ^b^
		16.14	43.53 ± 2.04 ^b^	54.12 ± 2.04 ^b^	64.71 ± 2.04 ^b^
		22.56	55.3 ± 1.18 ^b^	65.89 ± 1.18 ^b^	84.7 ± 2.35 ^b^
		29.03	72.94 ± 5.13 ^b^	62.35 ± 20.10 ^b^	98.82 ± 1.18 ^a^
		35.49	100.00 ± 0 a	100.00 ± 0 ^a^	100.00 ± 0 ^a^
	Spirodiclofen	7.20	27.71 ± 5.52 ^a^	29.49 ± 4.62 ^b^	36.99 ± 3.62 ^b^
		10.80	32.53 ± 3.19 ^a^	39.74 ± 4.62 ^ab^	53.42 ± 5.48 ^ab^
		14.40	33.73 ± 8.69 ^a^	43.59 ± 10.49 ^ab^	53.42 ± 3.62 ^ab^
		18.00	40.96 ± 2.41 ^a^	58.97 ± 7.8 ^ab^	64.38 ± 14.50 ^ab^
		21.53	48.19 ± 3.19 ^a^	62.82 ± 3.39 ^a^	78.08 ± 3.62 ^a^
	Cyflumetofen	10.80	15.66 ± 1.21 ^b^	32.05 ± 6.79 ^bc^	46.58 ± 8.55 ^bc^
		12.60	13.25 ± 2.09 ^b^	16.66 ± 1.28 ^c^	23.29 ± 5.97 ^c^
		14.40	34.94 ± 2.08 ^ab^	46.15 ± 2.22 ^abc^	49.31 ± 3.62 ^bc^
		16.20	45.78 ± 12.52 ^a^	61.54 ± 13.33 ^ab^	89.04 ± 7.25 ^a^
		18.00	56.63 ± 3.62 ^a^	71.80 ± 6.79 ^a^	76.71 ± 8.33 ^ab^
Others	Buprofezin	5.18	7.14 ± 2.06 ^b^	33.73 ± 3.19 ^a^	50.63 ± 4.39 ^c^
		5.92	25.00 ± 4.12 ^ab^	34.94 ± 6.26 ^a^	54.43 ± 5.80 ^c^
		6.66	32.14 ± 7.43 ^a^	39.76 ± 6.71 ^a^	69.62 ± 4.39 ^bc^
		7.4	46.43 ± 3.57 ^a^	57.83 ± 7.33 ^a^	83.54 ± 2.53 ^ab^
		8.15	45.24 ± 7.24 ^a^	62.65 ± 6.38 ^a^	92.41 ± 2.19 ^a^
	Flonicamid	4.5	20.48 ± 8.35 ^b^	29.49 ± 3.39 ^c^	36.99 ± 3.62 ^c^
		5.22	36.14 ± 4.34 ^b^	42.31 ± 5.87 ^bc^	50.68 ± 2.37 ^bc^
		5.94	34.94 ± 2.08 ^b^	38.46 ± 2.22 ^bc^	50.68 ± 6.27 ^bc^
		6.66	43.37 ± 6.37 ^ab^	50.00 ± 3.85 ^b^	60.27 ± 2.74 ^b^
		7.38	67.47 ± 2.08 ^a^	69.23 ± 2.22 ^a^	86.30 ± 3.62 ^a^
	Pymetrozine	14.85	22.89 ± 1.21 ^a^	23.08 ± 2.22 ^a^	28.77 ± 3.62 ^a^
		29.7	31.33 ± 9.56 ^a^	30.77 ± 10.18 ^a^	42.47 ± 16.61 ^a^
		45	40.96 ± 7.33 ^a^	43.59 ± 7.80 ^a^	54.80 ± 12.55 ^a^
		59.85	34.94 ± 7.52 ^a^	37.18 ± 7.80 ^a^	41.10 ± 7.63 ^a^
		74.7	33.74 ± 6.37 ^a^	38.46 ± 4.44 ^a^	52.05 ± 3.62 ^a^
	Pyriproxyfen	3.78	32.53 ± 9.41 ^a^	37.18 ± 11.18 ^a^	53.43 ± 13.07 ^a^
		4.68	21.69 ± 11.49 ^a^	24.36 ± 10.01 ^a^	39.73 ± 2.74 ^a^
		5.58	39.76 ± 4.34 ^a^	43.59 ± 6.41 ^a^	56.16 ± 3.62 ^a^
		6.48	37.35 ± 10.71 ^a^	42.31 ± 14.56 ^a^	60.27 ± 10.7 ^a^
		7.38	43.37 ± 8.69 ^a^	47.44 ± 9.25 ^a^	69.86 ± 11.7 ^a^

Note: The values in the table are the mean ± SE of each dose on each day of assessment. One-way ANOVA was used to determine significant differences among treatment groups. For the same insecticide, different lowercase letters following values in the same column indicate significant differences between the doses according to Tukey’s test (*p* < 0.05). Significance is indicated in the following tables in the same way.

**Table 3:** Field efficacy of imidacloprid, fenthion, sulfoxaflor, and cyetpyrafen against *Thrips flavus* (Changchun, Jilin Province, 2023).

Insecticides	Dose (g a.i.·hm^−2^)	Field Efficacy (%)			
		Days After Application (day)			
		1	3	7	14
Imidacloprid	2.25	45.76 ± 9.74 ^a^	46.10 ± 7.71 ^b^	57.88 ± 3.49 ^b^	39.68 ± 13.47 ^a^
	4.50	61.37 ± 5.25 ^a^	62.12 ± 4.27 ^ab^	78.04 ± 3.71 ^ab^	62.68 ± 2.90 ^a^
	6.75	59.18 ± 7.52 ^a^	63.09 ± 2.27 ^ab^	60.64 ± 6.22 ^ab^	55.44 ± 11.18 ^a^
	9.00	62.27 ± 5.58 ^a^	68.19 ± 6.16 ^ab^	68.04 ± 7.50 ^ab^	58.25 ± 9.96 ^a^
	11.25	74.57 ± 1.31 ^a^	77.31 ± 4.97 ^a^	82.91 ± 3.63 ^a^	52.34 ± 19.30 ^a^
Fenthion	2.25	63.10 ± 7.55 ^a^	64.32 ± 3.26 ^a^	68.28 ± 3.48 ^a^	60.92 ± 6.46 ^a^
	4.50	66.15 ± 10.07 ^a^	72.10 ± 1.82 ^a^	67.08 ± 1.42 ^a^	49.87 ± 8.66 ^a^
	6.75	60.81 ± 11.81 ^a^	59.26 ± 11.90 ^a^	69.98 ± 9.32 ^a^	33.71 ± 6.91 ^a^
	9.00	71.14 ± 6.40 ^a^	68.31 ± 4.18 ^a^	61.9 ± 8.75 ^a^	58.71 ± 12.08 ^a^
	11.25	67.99 ± 8.77 ^a^	78.65 ± 8.18 ^a^	84.23 ± 2.05 ^a^	41.88 ± 4.06 ^a^
Sulfoxaflor	0.40	66.97 ± 7.50 ^a^	69.78 ± 4.86 ^a^	70.64 ± 7.07 ^a^	29.07 ± 21.53 ^a^
	0.59	69.06 ± 6.41 ^a^	67.73 ± 8.40 ^a^	67.77 ± 9.14 ^a^	38.61 ± 10.80 ^a^
	0.79	71.26 ± 4.46 ^a^	69.74 ± 9.79 ^a^	72.49 ± 5.28 ^a^	40.33 ± 6.98 ^a^
	0.99	42.51 ± 11.12 ^a^	67.69 ± 2.89 ^a^	63.39 ± 7.52 ^a^	21.40 ± 9.85 ^a^
	1.19	69.23 ± 2.94 ^a^	73.35 ± 3.74 ^a^	80.23 ± 2.08 ^a^	37.40 ± 11.48 ^a^
Cyetpyrafen	0.90	59.88 ± 5.04 ^a^	64.77 ± 4.61 ^a^	64.80 ± 5.61 ^a^	35.48 ± 11.85 ^a^
	2.70	53.91 ± 11.94 ^a^	65.14 ± 1.79 ^a^	41.99 ± 14.76 ^a^	32.78 ± 8.05 ^a^
	4.51	66.35 ± 2.28 ^a^	59.68 ± 7.57 ^a^	53.89 ± 7.27 ^a^	39.23 ± 12.25 ^a^
	6.29	58.46 ± 4.83 ^a^	62.37 ± 5.66 ^a^	53.43 ± 13.17 ^a^	30.13 ± 17.69 ^a^
	8.10	79.88 ± 1.67 ^a^	83.61 ± 5.36 ^a^	85.68 ± 0.44 ^a^	44.63 ± 14.37 ^a^

Note: The values in the table are mean ± SE. One-way ANOVA was used to determine significant differences among treatment groups. For the same insecticide, different lowercase letters following values in the same column indicate significant differences between the doses according to Tukey’s test (*p* < 0.05).

## Data Availability

The original contributions presented in this study are included in the article/Supplementary Materials. Further inquiries can be directed to the corresponding author.

## Supplementary Materials

The following supporting information is available for download at https://www.mdpi.com/article/10.3390/insects16040405/s1. Table S1: Basic information of the thirty insecticides evaluated against *Thrips flavus*; Table S2: Concentration gradients of the thirty insecticides in laboratory bioassay; Table S3. Pot experiment Concentration gradients of thirty insecticides in the pot experiment; Table S4: The insecticide efficacy of the thirty insecticides at the highest concentrations; Figure S1: Schematic diagram of the field experiment; Figure S2: Insecticide efficacy of the thirty insecticides against *T*. *flavus*.
