# Supplementary material for: Toxicity and Efficacy of Thirty Insecticides Against Thrips flavus in Northeast China: Laboratory, Semifield, and Field Trials
Source: Insects. 2025 Apr 11;16(4):405. doi: 10.3390/insects16040405 (PMC12027959; doi:10.3390/insects16040405)
Supplement: Supplementary file 1 [file insects-16-00405-s001.zip › insects-3533939-supplementary.pdf]

Supplementary Materials

Table S1. Basic information of thirty insecticides evaluated against *Thrips flavus*.

Insecticide trade name	Common name	Formulation	Group	WHO hazard classification	IRAC group	Mode of action	Registration of active ingredients on thrips	Commonly recommended dosage （g·hm^{-2}）	Manufacturer
Acetamiprid 5% EC	Acetamiprid	5% EC	Neonicotinoids	Class II	4A	Nicotinic acetylcholine receptor (nAChR) competitive modulators	yes	216	Hebei Nongxin Biological Technology Co., Ltd., China
Imidacloprid 25% WP	Imidacloprid	25% WP	Neonicotinoids	Class II	4A	Nicotinic acetylcholine receptor (nAChR) competitive modulators	yes	90	Hebei Kaiste Agrochemical Co., Ltd. China
Dinotefuran 20% SC	Dinotefuran	20% SC	Neonicotinoids	Class III	4A	Nicotinic acetylcholine receptor (nAChR) competitive modulators	yes	600	Guangxi Guilin Hongtian Biochemical Co., Ltd. China
Nitenpyram 20% WG	Nitenpyram	20% WG	Neonicotinoids	Class II	4A	Nicotinic acetylcholine receptor (nAChR) competitive modulators	no	375 (rice planthopper)	Shanxi Huarong Kaiwei Biological Co., Ltd. China
Thiacloprid 40%SC	Thiacloprid	40% SC	Neonicotinoids	Class II	4A	Nicotinic acetylcholine receptor (nAChR) competitive modulators	yes	210	Limin Chemical Co., Ltd., China
Sulfoxaflor 22% SC	Sulfoxaflor	22% SC	Neonicotinoids	Class II	4C	Nicotinic acetylcholine receptor (nAChR) competitive modulators	no	300 (aphid)	Corteva Agriscience Inc., USA
lambda-Cyhalothrin 5% ME	lambda-Cyhalothrin	5% ME	Pyrethroids	Class II	3A	Sodium channel modulators	yes	180	Hebei Zhongbao Green Crops Technology Co., Ltd., China
beta-Cypermethrin 4.5% EC	beta-Cypermethrin	4.5% EC	Pyrethroids	—	3A	Sodium channel modulators	no	600 (*Artogeia rapae*)	Hebei Zhongbao Green Crops Technology Co., Ltd., China
Fenpropathrin 20% EC	Fenpropathrin	20% EC	Pyrethroids	Class II	3A	Sodium channel modulators	no	450 (*Artogeia rapae*)	Zhejiang Welldone Chemical Co., Ltd., China
Tetrachlorantraniliprole 10% SC	Tetrachlorantraniliprole	10% SC	Diamides	—	—	Ryanodine receptor modulators	no	600 (*Spodoptera exigua*)	Shenyang Sciencreat Chemicals Co. Ltd., China
Chlorantraniliprole 200g/L SC	Chlorantraniliprole	200 g/L SC	Diamides	Class U	28	Ryanodine receptor modulators	no	225 (*Spodoptera frugiperda*)	FMC Corporation, USA
Cyantraniliprole 30% OD	Cyantraniliprole	30% OD	Diamides	Class U	28	Ryanodine receptor modulators	yes	300	FMC Corporation, USA
Tetraniliprole 200 g/L SC	Tetraniliprole	200 g/L SC	Diamides	—	28	Ryanodine receptor modulators	no	225 (*Spodoptera frugiperda*)	Bayer CropScience (China) Co., Ltd., China
Monosultap 90% SP	Monosultap	90% SP	Nereistoxins	—	—	Nicotinic acetylcholine receptor (nAChR) channel blockers	yes	660	Anhui Huaxing Chemical Industry Co., Ltd., China
Cartap 98% SP	Cartap	98% SP	Nereistoxins	Class II	14	Nicotinic acetylcholine receptor (nAChR) channel blockers	yes	37500	Sumitomo Chemical Co., Ltd., Japan
Bisultap 25% AS	Bisultap	25% AS	Nereistoxins	—	—	Nicotinic acetylcholine receptor (nAChR) channel blockers	no	3750 (*Chilo suppressalis*)	Anhui Huaxing Chemical Industry Co., Ltd., China
Fenthion 50% EC	Fenthion	50% EC	organophosphates	Class II	1B	Acetylcholinesterase (AChE) inhibitors	yes	225	Guangdong Foshan Yinghui Crop Science Co., Ltd., China

Malathion 45% EC	Malathion	45% EC	organophosphates	Class III	1B	Acetylcholinesterase (AChE) inhibitors	yes	1650	Hebei Jindelun Biochemical Technology Co., Ltd., China
Cyflumetofen 20% SC	Cyflumetofen	20% SC	Beta-ketonitrile derivatives	Class III	25A	Mitochondrial complex II electron transport inhibitors	no	563 (leaf mite)	Jiangsu FMC Plant Protection Co., Ltd., China
Cyetpyrafen 30% SC	Cyetpyrafen	30% SC	Beta-ketonitrile derivatives	—	—	Mitochondrial complex II electron transport inhibitors	no	150 (leaf mite)	Shenyang Sciencreat Chemicals Co. Ltd., China
Cyenopyrafen 30% SC	Cyenopyrafen	30% SC	beta-ketonitrile derivatives	—	25A	Mitochondrial complex II electron transport inhibitors	no	375 (*Tetranychus urticae*)	Nissan Chemical Corporation, Japan
Pyridaben 15% EC	Pyridaben	15% EC	pyridazinone	Class II	21A	Mitochondrial complex I electron transport inhibitors	yes	1800	Nanjing Red Sun Co., Ltd., China
Bifenazate 43% SC	Bifenazate	43% SC	Biphenylhydrazines	Class U	20D	Mitochondrial complex III electron transport inhibitors – Qo site	no	375 (*Tetranychus urticae*)	Arista Biochemicals Co., Ltd, Japan
Spirodiclofen 24% SC	Spirodiclofen	24% SC	Tetronic And Tetramic Acid Derivatives	Class III	23	Inhibitors of acetyl-CoA carboxylase	no	187 (red spider)	Hebei Zhongbao Green Crops Technology Co., Ltd., China
Spirotetramat 22.4% SC	Spirotetramat	22.4% SC	Tetronic And Tetramic Acid Derivatives	Class III	23	Inhibitors of acetyl-CoA carboxylase	yes	450	Bayer AG, Germany
Chlorfenapyr 10% SC	Chlorfenapyr	10% SC	Pyrroles	Class II	13	Uncouplers of oxidative phosphorylation via disruption of the proton gradient	yes	451	BASF Societas Europaea, Germany
Pymetrozine 50% WG	Pymetrozine	50% WG	Pyridine Azomethine Derivatives	Class III	9B	Chordotonal Organ TRPV Channel Modulators	yes	615	Syngenta Nantong Crop Protection Co., Ltd., China
Pyriproxyfen 100 g/L EC	Pyriproxyfen	100 g/L EC	Juvenile Hormone Analogues	Class U	7C	Juvenile hormone receptor modulators	yes	450	Shanghai Biochemical Agricultural Products Co., Ltd., China
Flonicamid 10% WG	Flonicamid	10% WG	Pyridinecarboxamide	Class II	29	Chordotonal organ nicotinamidase inhibitors	yes	210	Ishihara Sangyo Kaisha, Ltd., Japan
Buprofezin 25% WP	Buprofezin	25% WP	Benzothiadiazines	Class III	16	Inhibitors of chitin biosynthesis, type 1	no	450 (rice planthopper)	Adama Anbang (Jiangsu) Co., Ltd., China

Note: In this table, SC: suspension concentrate; WG: water soluble concentrate; EC: emulsifiable concentrate; SP: water soluble powder; WP: wettable powder; AS: aqueous solutions; ME: micro-emulsion; OD: oil-based suspension concentrate. "—" means that no information was found for the corresponding item.

Table S2. The concentration gradients of thirty insecticides in laboratory bioassay

Number	Insecticides	Concentration gradient (mg/L)				
		I	II	III	IV	V
1	Cyflumetofen	60.0	70.0	80.0	90.0	100.0
2	Cyetpyrafen	3.3	10.0	16.7	23.3	30.0
3	Pyridaben	11.1	22.2	33.3	44.4	55.5
4	Chlorfenapyr	10.0	20.0	30.0	40.0	50.0
5	Cyenopyrafen	10.0	20.0	30.0	40.0	50.0
6	Spirodiclofen	33.3	50.0	66.7	83.3	99.7
7	Spirotetramat	8.0	16.0	24.0	32.0	40.0
8	Buprofezin	23.0	26.3	29.6	32.9	36.2
9	Bifenazate	25.0	41.7	58.3	75.0	91.7
10	Acetamiprid	20.0	40.0	60.0	80.0	100.0
11	Imidacloprid	10.0	20.0	30.0	40.0	50.0
12	Dinotefuran	38.0	41.0	45.0	48.0	51.0

13	Sulfoxaflor	2.0	3.0	4.0	5.0	6.0
14	Thiacloprid	116.6	133.3	150.0	166.6	183.3
15	Nitenpyram	16.0	33.0	50.0	66.0	83.0
16	Lambda-Cyhalothrin	16.6	33.3	50.0	66.6	83.3
17	Beta-Cypermethrin	33.3	50.0	66.7	83.3	99.7
18	Fenpropathrin	16.0	19.0	22.0	25.0	28.0
19	Monosultap	50.0	66.0	83.0	100.0	116.0
20	Cartap	50.0	66.0	83.0	100.0	116.0
21	Bisultap	20.0	40.0	60.0	80.0	100.0
22	Fenthion	5.0	10.0	15.0	20.0	25.0
23	Malathion	10.0	18.0	26.0	34.0	42.0
24	Pymetrozine	33.0	66.0	100.0	133.0	166.0
25	Tetrachlorantraniliprole	8.3	16.7	25.0	33.3	41.7
26	Chlorantraniliprole	6.7	13.3	20.0	26.7	33.3
27	Cyantraniliprole	10.0	20.0	30.0	40.0	50.0

28	Tetraniliprole	20.0	40.0	60.0	80.0	100.0
29	Pyriproxyfen	42.0	52.0	62.0	72.0	82.0
30	Flonicamid	50.0	58.0	66.0	74.0	82.0

Table S3. Pot experiment concentration gradient of thirty insecticides.

Number	Insecticides	Concentration gradient (g a.i.·hm^{-2})				
		I	II	III	IV	V
1	Cyflumetofen	10.80	12.60	14.40	16.20	18.00
2	Cyetpyrafen	0.90	2.70	4.51	6.29	8.10
3	Pyridaben	1.50	3.00	4.50	5.99	7.49
4	Chlorfenapyr	0.90	1.80	2.70	3.60	4.50
5	Cyenopyrafen	2.70	5.40	8.10	10.80	13.50
6	Spirodiclofen	7.20	10.80	14.40	18.00	21.53
7	Spirotetramat	1.61	3.23	4.84	6.45	8.06
8	Buprofezin	5.18	5.92	6.66	7.40	8.15
9	Bifenazate	9.68	16.14	22.56	29.03	35.49
10	Acetamiprid	0.90	1.80	2.70	3.60	4.50
11	Imidacloprid	2.25	4.50	6.75	9.00	11.25

12	Dinotefuran	6.84	7.38	8.10	8.64	9.18
13	Sulfoxaflor	0.40	0.59	0.79	0.99	1.19
14	Thiacloprid	41.98	47.99	54.00	59.98	65.99
15	Nitenpyram	2.88	5.94	9.00	11.88	14.94
16	Lambda-Cyhalothrin	0.75	1.50	2.25	3.00	3.75
17	Beta-Cypermethrin	1.35	2.03	2.70	3.37	4.04
18	Fenpropathrin	2.88	3.42	3.96	4.50	5.04
19	Monosultap	40.50	53.46	67.23	81.00	93.96
20	Cartap	44.10	58.21	73.21	88.20	102.31
21	Bisultap	4.50	9.00	13.50	18.00	22.50
22	Fenthion	2.25	4.50	6.75	9.00	11.25
23	Malathion	4.05	7.29	10.53	13.77	17.01
24	Pymetrozine	14.85	29.70	45.00	59.85	74.70
25	Tetrachlorantraniliprole	0.75	1.50	2.25	3.00	3.75
26	Chlorantraniliprole	1.21	2.39	3.60	4.81	5.99

27	Cyantraniliprole	2.70	5.40	8.10	10.80	13.50
28	Tetraniliprole	3.60	7.20	10.80	14.40	18.00
29	Pyriproxyfen	3.78	4.68	5.58	6.48	7.38
30	Flonicamid	4.50	5.22	5.94	6.66	7.38

Table S4. The insecticide efficacy of thirty insecticides against *Thrips flavus* at the highest concentrations.

Number	Insecticides	Highest concentration (g a.i.·hm^{-2})	Insecticide efficacy (%)		
			After 1 d of application	After 3 d of application	After 7 d of application
1	Fenthion	11.25	71.09±3.61bcde	84.62±3.85abcdefg	100.00a
2	Sulfoxaflor	1.19	89.16±5.52ab	97.44±2.56ab	100.00a
3	Cyetpyrafen	8.10	80.95±2.38bcd	91.67±3.15abcd	98.81±1.19ab
4	Imidacloprid	11.25	38.1±3.15efghi	53.01±5.52ghijk	98.73±1.27ab
5	Cyenopyrafen	13.5	59.03±11.49cdefgh	79.49±10.01bcdefghi	90.41±3.62abcdefg
6	Malathion	17.01	65.06±4.34cdefg	94.87±2.56abc	100.00a
7	Fenpropathrin	5.04	66.26±3.19bcdef	78.21±4.62bcdefghi	97.26±2.74abc
8	Chlorantraniliprole	5.99	87.06±3.11bc	91.76±3.11abcd	100.00a
9	Tetrachlorantraniliprole	3.75	42.17±5.52efghi	44.87±6.78ijk	56.16±9.88gh
10	Chlorfenapyr	4.50	69.88±3.19bcde	88.46±2.22abcdef	92.29±2.43abcdef

11	Cyantraniliprole	13.50	20.48±10.84i	23.08±9.68k	56.16±3.62h
12	Spirotetramat	8.06	45.78±4.17efghi	60.26±11.18defghijk	87.67±6.28abcdef
13	Lambda-Cyhalothrin	3.75	40.96±4.34efghi	55.13±3.39fghijk	89.04±5.48abcdef
14	Tetraniliprole	5.99	42.17±6.26efghi	56.41±12.23efghijk	67.12±9.49efgh
15	Pyridaben	7.49	82.93±3.22bcd	90.24±3.23abcde	96.34±2.11abcd
16	Nitenpyram	14.94	59.04±1.2cdefgh	73.08±4.44cdefghij	83.56±2.37abcdefgh
17	Bifenazate	35.49	100.00a	100.00a	100.00a
18	Buprofezin	8.15	45.24±7.24efghi	62.65±6.38defghij	92.41±2.19abcdef
19	Dinotefuran	9.18	46.43±5.46efghi	59.04±10.5defghijk	93.67±3.35abcdef
20	Bisultap	22.5	32.53±1.2ghi	43.59±5.59ijk	82.19±7.63bcdefgh
21	Acetamiprid	4.50	67.47±2.08bcde	83.34±1.28bcdefgh	94.44±1.11abcdef
22	Pymetrozine	74.70	33.74±6.37fghi	38.46±4.44jk	52.05±3.62h
23	Flonicamid	7.38	67.47±2.08bcde	69.23±2.22cdefghij	86.3±3.62abcdefgh
25	beta-Cypermethrin	4.04	30.12±8.69hi	51.1±10.17ghijk	94.52±2.74abcde
26	Cartap	102.31	59.04±1.2cdefgh	65.38±2.22defghij	71.23±4.75defgh

27	Cyflumetofen	18.00	56.63±3.62defgh	71.8±6.79cdefghij	76.71±8.33cdefgh
28	Spirodiclofen	21.53	48.19±3.19efghi	62.82±3.39defghij	78.08±3.62cdefgh
24	Pyriproxyfen	7.38	43.37±8.69efghi	47.44±9.25hijk	69.86±11.7defgh
29	Monosultap	93.96	56.63±5.52defgh	60.26±4.62defghijk	67.12±2.37efgh
30	Thiacloprid	65.99	55.42±4.82defgh	62.65±4.34defghij	69.86±4.94efgh

Note: Different lowercase letters following numbers in the same column indicate significant differences between the two treatments ($p < 0.05$).

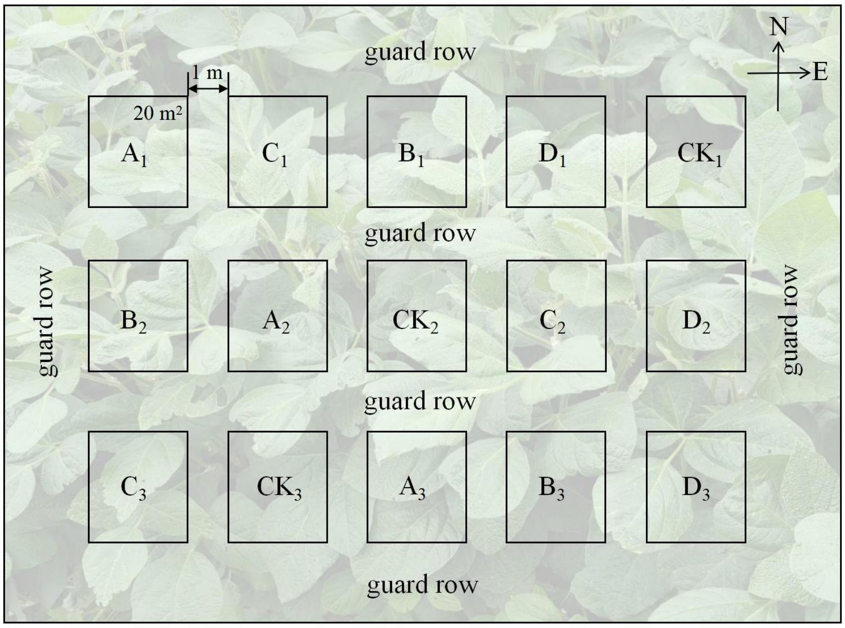

Figure S1. Schematic diagram of the field experiment.

Note: A_1, A_2 and A_3 are three replications of the imidacloprid treatment group; B_1, B_2 and B_3 are three replications of malathion treatment group; C_1, C_2 and C_3 are three replications of the sulfoxaflor treatment group; D_1, D_2 and D_3 are three replications of the cyetpyrafen treatment group; CK is the blank control group. Only three replicates of one concentration of each insecticide were indicated in the schematic.

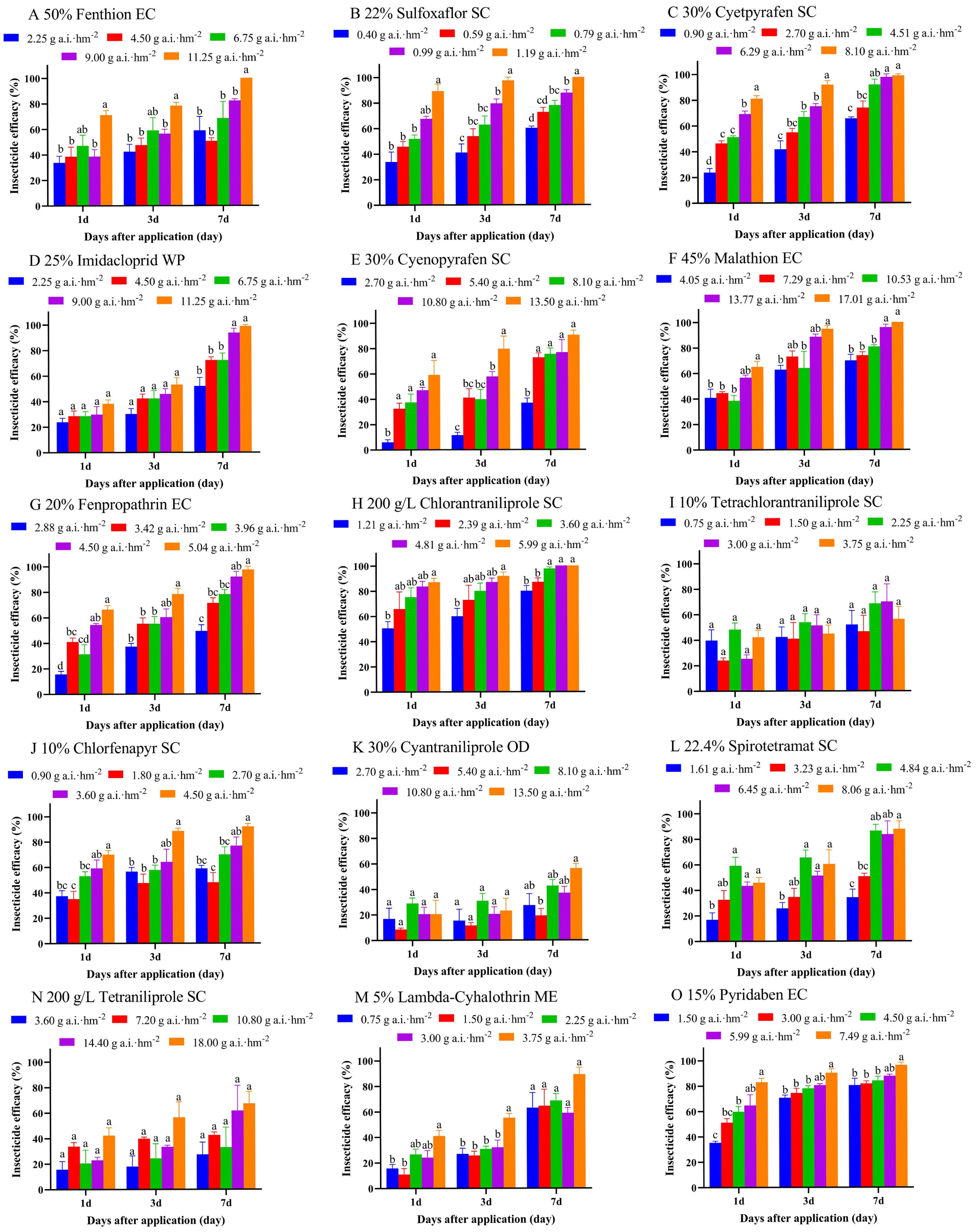

Figure S2. Insecticide efficacy of thirty chemical insecticides against *Thrips flavus*.

Note: Different lowercase letters on each column indicate that there are significant differences in different concentrations ($p < 0.05$).

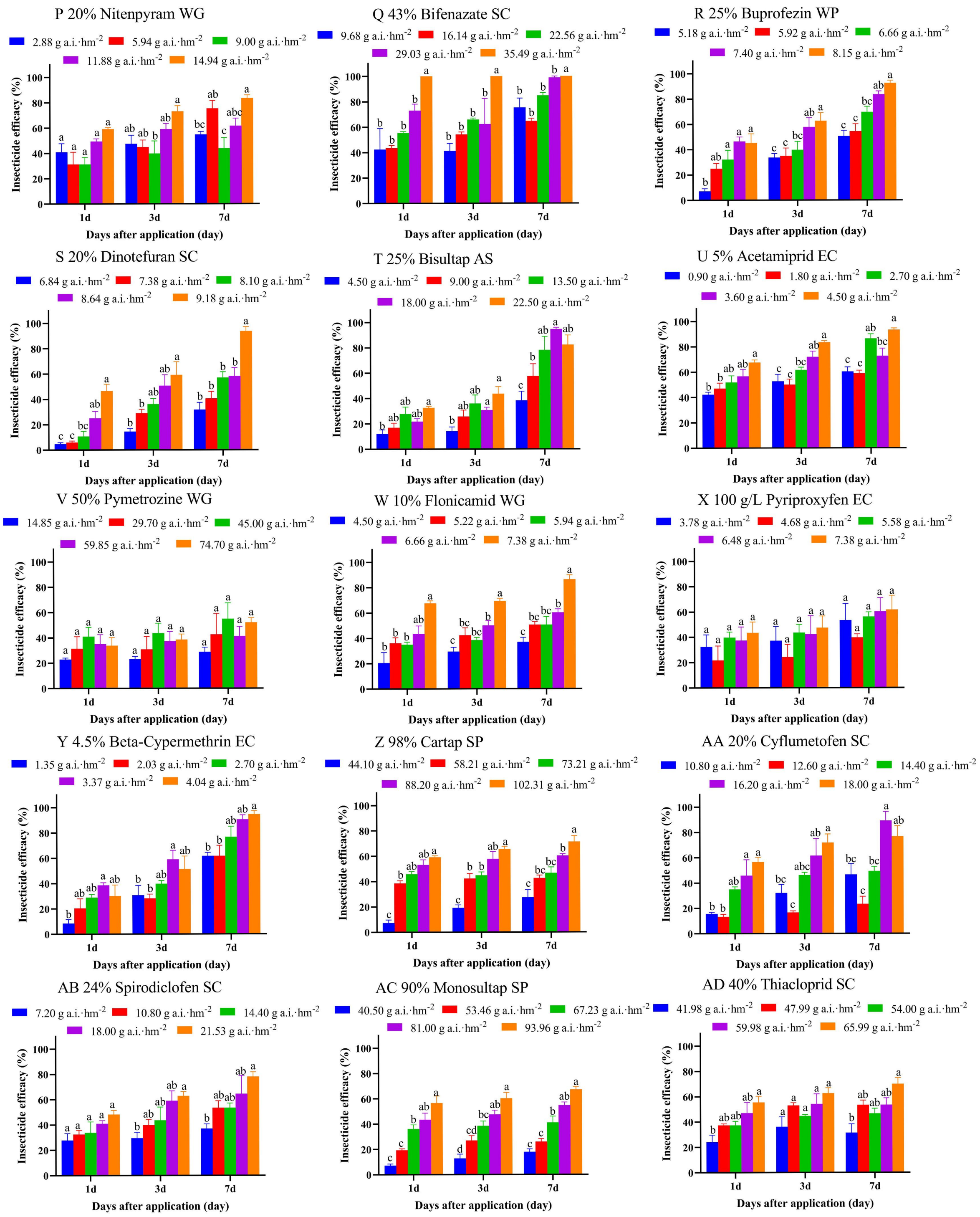

Figure S2. Insecticide efficacy of thirty chemical insecticides against *Thrips flavus*.

Note: Different lowercase letters on each column indicate that there are significant differences in different concentrations ($p < 0.05$).
